# Characterization and potential diagnostic application of monoclonal antibodies specific to rabies virus^☆^

**DOI:** 10.1016/S1674-8301(10)60053-X

**Published:** 2010-09

**Authors:** Xinjian Liu, Xiaomin Feng, Qi Tang, Zhongcan Wang, Zhenning Qiu, Yuhua Li, Changjun Wang, Zhenqing Feng, Jin Zhu, Xiaohong Guan

**Affiliations:** a Key Laboratory of Antibody Technique of Ministry of Health, Nanjing Medical University, Nanjing 210029, Jiangsu Province, China; b Huadong Medical Institute of Biotechniques, Nanjing 210002, Jiangsu Province, China

**Keywords:** rabies, monoclonal antibody, purified antibody, immunofluorescence, immunocytochemistry, antigen capture-ELISA

## Abstract

**Objective:**

Rabies is invariably a fatal encephalomyelitis that is considered to be a serious public health problem. It is necessary to develop standard rabies virus diagnostic tools, especially for diagnosing the strains prevalent in China.

**Methods:**

Monoclonal antibodies (MAbs) specific to rabies virus were produced and characterized by enzyme linked immunosorbent assay (ELISA), isotyping, affinity assay, immunofluorescence assay (IFA), and immunocytochemistry. The MAb, whose affinity was higher for antigen, was used to establish an antigen capture-ELISA (AC-ELISA) detection system and test the efficiency by using clinical samples.

**Results:**

The heavy chain subclasses of two MAbs were all determined to be IgG2a. The 3C7 MAb showed stronger reactivity with rabies virus protein than the 2C5 MAb in an ELISA analysis, whereas the 3C7 MAb showed the highest affinity for antigen. IFA and immunocytochemistry results also indicated that the two MAbs could recognize rabies virus protein in its native form in cell samples. Data obtained using clinical samples showed that rabies virus could be detected by AC-ELISA detection system using the 3C7 MAb.

**Conclusion:**

It was potentially useful for the further development of highly sensitive, easily handled, and relatively rapid detection kits/tools for rabies surveillance in those areas where rabies is endemic, especially in China.

## INTRODUCTION

Rabies is a viral encephalitis that is considered to be a reemerging zoonosis throughout much of the world[1]-[3]. Rabies virus (RABV) is a member of the genus *Lyssavirus*, within the family Rhabdoviridae[4]. The Rhabdoviridae are negative sense single-stranded RNA viruses with a distinctive bullet-shape structure[4]. Rabies is a major disease of public health affecting human beings as well as domestic and wild animals in many parts of the world, especially in many developing countries where it is endemic among dogs[5],[6]. Globally, more than 2.5 billion people live in regions where rabies is endemic. It is estimated that at least 14 million people receive post-exposure vaccinations, and 55,000 people die from rabies yearly[7],[8].

As the clinical signs and symptoms of rabies infection are non-specific compared to those cases of encephalitis caused by other agents, such as tetanus and several viral encephalitises, diagnosis relies mainly on laboratory tests. In the past, histopathological methods including the detection of Negri bodies[9], intracranial inoculation of mice with virus[10] and a latex agglutination test (LAT)[11] were used for the detection of rabies virus or antigen. But these methods are laborious and expensive, which limit their application in large-scale surveillance programs. In the most modern laboratories, rabies is usually diagnosed by the detection of viral antigen in the brain by using direct fluorescent antibody test[12]. Thus far, virus isolation methods have generally been performed to determine the presence of infectious virus in samples. The presence of rabies virus can also be detected by RT-PCR[13]. However, in some developing countries, especially in rural areas, RT-PCR is difficult to perform. Enzyme-linked immunosorbent assay (ELISA)-based detection for IgM, IgG or IgA has been developed, and some of these assays are commercially available. Detection of lyssavirus nucleocapsid antigen by ELISA has been described and used for many years in some laboratories[13],[14]. It is rapid and can be useful for epidemiological surveys. Nowadays, rabies rapid enzyme immuno-diagnosis (RREID) is being used widely[15], but microplates are coated with purified rabbit anti-rabies virus polyclonal antibody, which may affect the specificity of diagnosis. Additionally, at present this test is not commercially available[13].

In the present study the CTN strain of rabies virus (isolated from brain tissue of a patient with rabies from Zibo, Shandong Province, China) was chosen as the immunogen, as the structure of its glycoprotein (GP) gene is basically stable and the homology of the CTN strain to the street strain is higher than those of aG (isolated from a rabid dog's brain from Beijing, China) and Pasteur Virus (PV) strains in China[16]. We reported the production of two monoclonal antibodies (MAbs) against the rabies virus and the properties of the MAbs, which were determined by isotyping, affinity assay and immunocytochemistry. The results demonstrated that the MAbs could be applied to various analytical methods, such as IFA, dot-ELISA and antigen-capture ELISA, for diagnosis and functional studies of rabies protein, especially for the strains prevalent in China.

## MATERIALS AND METHODS

### Cell lines and virus strains

The cell line BHK-21 and the mouse myeloma cell line SP2/0 were routinely cultured in DMEM (Life Technologies, USA) supplemented with 10% fetal calf serum in a 37°C humidified incubator with a mixture of 95% air and 5% CO_2_. Rabies virus strains CTN and CVS-11 were provided by Wuhan Institute of Virology, Chinese Academy of Sciences. Rabies virus strain Flury and CVS-24 were obtained from the Veterinary Institute of the Academy of Military Medical Sciences, China.

### Immunogen and mice

The purity of inactivated rabies virus strain CTN was more than 99%. Six BALB/c mice, aged 6 w, were obtained from the Center of Comparative Medicine of Yangzhou University. All animal procedures were approved by the Ethical Committee on Animal Care of Nanjing Medical University. Kunming mice (10-12 g) were infected with 100 LD50/0.05 mL rabies virus.

### Immunization of mice and production of monoclonal antibody

Five BALB/c mice were primed by subcutaneous injections (20 µg/mouse) with inactivated rabies virus of the CTN strain, mixed with complete Freund's adjuvant (Sigma, USA). Two boosts were given at d 14 and 28, followed by 100 µg of the same antigens (20 µg/mouse) mixed with incomplete adjuvant (Sigma) at d 42. Three days after the last boost, the titer of polyclonal antiserum was determined using indirect ELISA (described below) with virus protein as the antigen[17].

The mouse with the highest titer (1:51,200) was chosen for harvesting splenocytes. The spleen cells (10^8^) were fused with the SP2/0 myeloma cells (10^7^) in the presence of 50% PEG solution (Sigma), according to a previously described protocol[18]. Positive hybrids were immediately subcloned successively 4 times by limiting dilution to ensure monoclonality and stability.

### Reactivity of MAbs with rabies protein in an indirect ELISA

Ninety-six-well plates (Costar, Sigma) were coated with the purified rabies virus protein (0.5 µg/well) diluted in 50 mmol/L carbonate saline (pH 9.6) and incubated overnight at 4°C. Plates were blocked by incubating for 2 h at 37°C using 1% BSA in PBS. After washing, serially diluted MAbs in PBS (100 µL) were added to each well in triplicate and incubated for 1 h at 37°C followed by three more washings with 0.5% Tween-20 in PBS (PBST). In all ELISA assays, anti-VEGFR MAb was used as an antibody control, and then washed with PBST three times. Goat anti-mouse IgG horseradish peroxidase (1:5,000, Santa Cruz Biotechnology, USA) was added, and the samples were incubated for 1 h at 37°C. After washing with PBST, 100 µL of the mixture of H_2_O_2_ and 3,3′5,5′-tetramethyl-benzidene substrate (TMB; Thermo Scientific, USA) was added to each well. The reaction was stopped by adding 50 µL of 2 mol/L H_2_SO_4_. Immunoreactivity was visualized by a Multiskan Spectrum spectrophotometer (Thermo Labsystems, USA). The positive threshold (cut-off value) was defined as twice the mean of the negative standard values[19].

### MAbs purification and isotype identification

Two positive cell lines (named 2C5 and 3C7) were used to generate ascites in BALB/c mice. Ascites were purified by protein G chromatography (GE, USA) according to a previously described protocol[21]. The isotypes were determined by a mouse monoclonal antibody isotyping kit (ISO-2KT, Sigma).

### Reactivity of MAbs with rabies protein in dot-ELISA

The dot-ELISA test and optimization studies were based on the method of Pappas *et al.*[20]. Briefly, a nitrocellulose membrane (Amersham, UK) was equilibrated for 1 h at 37°C with PBS, and then dried at 37°C. Purified inactivated CTN strain virus particles were diluted in PBS (pH 7.4) to a concentration of 5 µg/mL and dropped onto the nitrocellulose membrane (5 µL per point). Membranes were dried at 37°C in a drying box. After blocking overnight at 4°C with PBS containing 1% BSA (PBSA), the membranes were incubated for 2 h at 37°C with the two different purified ascites-derived MAbs 2C5 and 3C7 (2 µg/mL). As a negative control, an equal amount of purified anti-VEGFR MAb was treated in the same way. Samples were then washed 3 times with 0.05% PBST, and incubated with horseradish peroxidase conjugated goat anti-mouse IgG (1:2,000, Santa Cruz Biotechnology, USA) for 1 h at 37°C, and then washed again. Antibody binding was visualized by the addition of the mixture of H_2_O_2_ and 3,3′-diamino benzidine (DAB; Sigma). After incubation for 15 min at 37°C, the reaction was stopped by repeated washes in deionized water.

### Western blotting analysis of MAbs

Western blotting was performed as previously described[22]. Rabies virus particles of the CTN strain were run on 12% SDS-PAGE and electrotransferred onto a nitrocellulose membrane in transfer buffer. Lysate of BHK-21 cells, which are used routinely for rabies virus culture, was used in the same manner to act as a negative control. After the transfer, the membrane was washed in deionized water and blocked with 3% BSA in PBS at 37°C for 1 h. Membranes were then incubated for 2 h at 37°C with one of the two purified MAbs (2 µg/mL), washed 3 times in PBST and again incubated with a 1:2,000 dilution of goat anti-mouse IgG peroxidase conjugate for 1 h. Following further washes, bound antibodies were detected by the addition of the mixture of H_2_O_2_ and DAB, and after incubation for 15 min at 37°C, the reaction was stopped by washing with water.

### Affinity analysis by surface plasmon resonance (SPR)

Surface plasmon resonance (SPR) analysis was performed on a BIAcore T100 analytical system (GE)[23]. Rabies virus protein was diluted to 5 µg/mL with acetate buffer (10 mmol/L NaAc, pH 5.5, GE) and immobilized on the surface of a CM5 sensor chip (GE) to capture purified MAbs. Purified ascites fluid samples containing MAbs were diluted with HBS-EP buffer (GE) at concentrations ranging from 31.25 to 1,000 nmol/L and used at a constant flow rate of 30 µL/min for 3 min at 25 °C. The association time was 180 s and the dissociation time was 600 s, followed by regeneration with 50 mmol/L NaOH. The sensorgrams were evaluated using the BIAcore T100 evaluation software (GE).

### Immunofluorescence assay (IFA)

Binding of mouse ascites MAbs with rabies infected cells was determined by IFA. Sub-confluent BHK-21 cells, growing on coverslips in 24-well microplates with slides, were infected with the Flury strain of rabies. After incubation for 24 h, purified MAbs (1:100) were added to the virus-infected BHK-21 cells. After incubation at 37°C for 2 h, the coverslips were washed 3 times with PBST, and FITC-labeled anti-mouse IgG (Wuhan Boster Biological Technology, China) was added at a dilution of 1:50. Coverslips were washed again after 1 h incubation. The cell nuclei were stained with HOECHST 33342 solution (1:10,000, Dojindo, Japan) at 1 mg/mL for 5 min at 37°C, and the coverslips mounted and observed by fluorescence microscopy. Cells showing strong green fluorescence were recorded as positive. The uninfected BHK-21cells were used as negative control.

### Immunocytochemistry

The coverslips with BHK-21 cells infected with the Flury strain of rabies were used for immunocytochemistry. The cells were immobilized with ethanol. Endogenous peroxidase activity was quenched by 3% hydrogen peroxide in methanol for 30 min, and the samples were then blocked with 10% goat serum for 15 min and subsequently incubated with the hybridoma cell culture supernatant overnight at 4°C. After being washed extensively, samples were treated with biotinized goat anti-mouse antibody and then S-A/HRP (SP-9000 Histostain TM-Plus Kit, Zymed, ZSGB-BIO) for 20 min each. The reaction product was visualized with DAB exposure for 20 s. Counterstaining was performed with hematoxylin, and the coverslips were then dehydrated and mounted[24]. The uninfected BHK-21cells were used as negative control.

### Antigen capture-ELISA (AC-ELISA)

For the AC-ELISA, the purified MAbs (0.5 µg/well) diluted in 50 mmol/L carbonate saline (pH 9.6), were coated on wells for 3 h at 37°C. After blocking overnight at 4°C with PBS containing 5% non-fat dry milk, wells were washed 3 times with PBST. To determine the detection limit of the AC-ELISA, the CTN strain virus particles, serially diluted in PBS, were added to the wells (100 µL/well) and incubated for 3 h with BSA acting as negative control. For further identification of the AC-ELISA, culture supernatant (10^3.5^ TCID_50_/mL) of the Flurry strain of rabies and tissue suspensions from four mouse brain samples infected with rabies (CVS-11 strain, CVS-24 strain, and two samples from wild animals named ZSR and BJ, no identification) were diluted in PBS containing 1% non-fat dry milk (PBSM), added to the wells (100 µL/well) and incubated for 2 h. Purified CTN strain virus (50 ng/100 µL) and cell culture supernatant of canine parvovirus were used as positive and negative controls, respectively. After washing, 100 µL horseradish peroxidase-conjugated 3C7 detector MAb (1:500) was added to each well and incubated for 1 h at 37°C, and detected as above described[30]. The experiments that involved the use of rabies strains were performed in a BSL-3 laboratory (Veterinary Institute of the Academy of Military Medical Sciences, China).

## RESULTS

### Generation and purification of MAbs against rabies protein

**Fig. 1:**
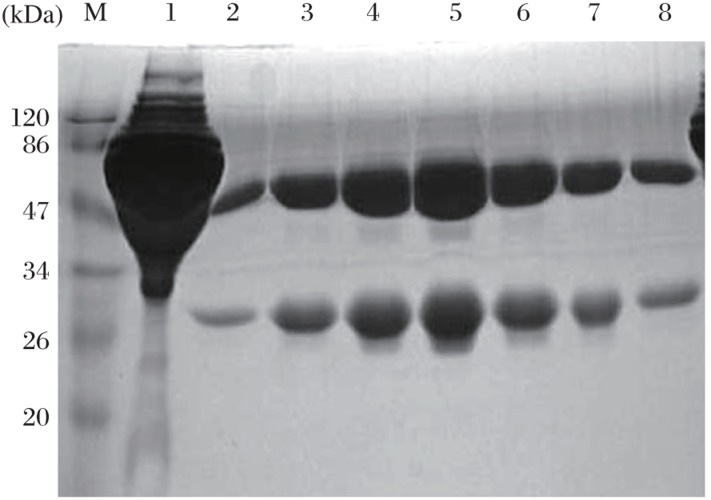
SDS-PAGE analysis of purified 3C7 MAb. Proteins were separated by 10% SDS/PAGE and stained with Coomassie Brilliant Blue. Lane 1 contained eluted flowthrough. Lanes 2-8 contained eluted protein with different concentrations of washing buffer cation, and the concentrations of the purified MAbs were in the range of 10-20 mg/mL. Lane M contained molecular-size markers.

The positive fused cell clones were screened using indirect ELISA with rabies virus protein as antigen. Hybridomas with higher ELISA titers were selected for screening, and two MAbs, 2C5 and 3C7, were finally isolated and cloned. Ascites was produced in BALB/c mice by the hybridomas. The heavy chain subclasses of the MAbs were all determined to be IgG2a. All ascites were efficiently purified by protein G chromatography. ***Fig. 1*** illustrates the results of SDS-PAGE of purified 3C7 MAb.

### Reactivity of MAbs with rabies protein in an indirect ELISA

To examine the reactivity of the MAbs with rabies virus protein, indirect ELISAs were performed using rabies protein. The titers of the two purified ascites MAbs were higher than 1:12,800 in indirect ELISA (data not shown). MAb 3C7 showed stronger positive binding to rabies protein over a greater range of dilutions than did MAb 2C5. Neither MAb showed significant binding to the negative control (SP2/0 culture supernatant).

### Dot-ELISA and Western-blotting analyses

**Fig. 2:**
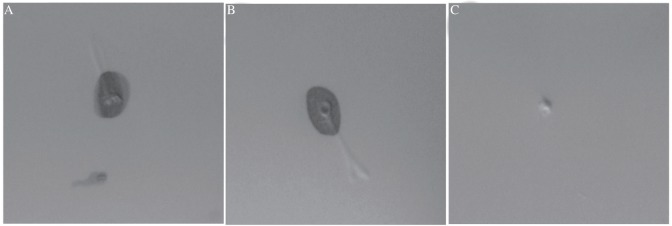
Dot-ELISA analysis of anti-rabies virus MAbs with rabies virus antigen. A: Antigen with 2C5 MAb. B: Antigen with 3C7 MAb. C: Antigen with anti-VEGFR MAb.

**Fig. 3:**
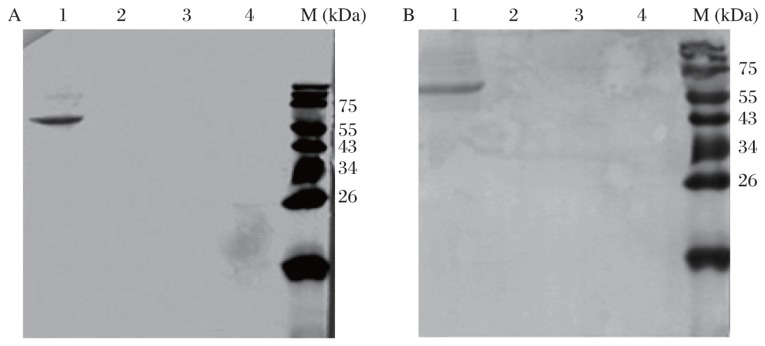
Western blot analysis of anti-rabies virus MAbs with antigen. A: 2C5 MAb with antigen. B: 3C7 MAb with antigen. Lane 1: rabies virus particles of the CTN strain. Lanes 2-4: BHK-21 cell lysates.

The binding specificities of the two MAbs against rabies protein were assessed by dot-ELISA (***Fig. 2***) and Western-blotting analyses (***Fig. 3***). The two MAbs bound to rabies virus proteins, allowing us to conclude that the two MAbs could recognize the rabies protein.

### Binding affinity between purified MAbs and rabies virus protein

**Fig. 4:**
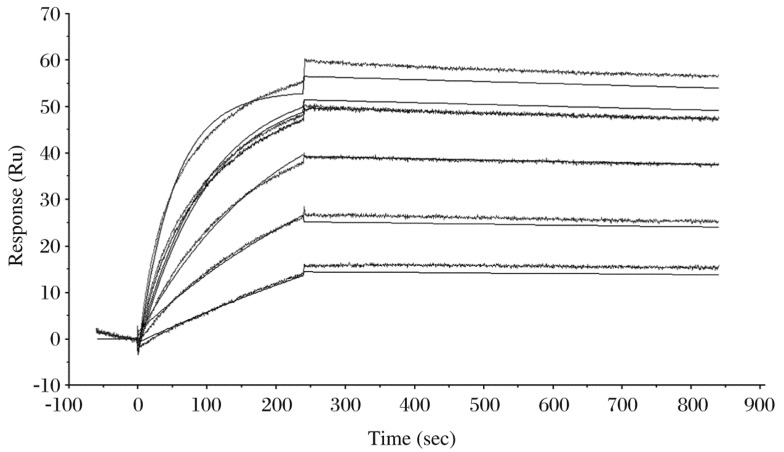
Biacore binding curves of purified MAbs with rabies virus protein. A concentration series from 31.25 to 1,000 nmol/L of purified 3C7 Mabs was injected (240 µL, associated for 4 min and then dissociated over 10 min). The affinity constant KD was determined as Koff/Kon.

The binding affinities between rabies protein and purified MAbs 2C5 and 3C7 were analyzed by the Biacore T100 system (GE). MAb 2C5 bound with an affinity of 3.644×10^−7^ mol/L (Figure not shown), while MAb 3C7 bound to rabies protein with an affinity of 3.272×10^−10^ mol/L (***Fig. 4***).

### Immunofluorescence assay (IFA)

**Fig. 5:**
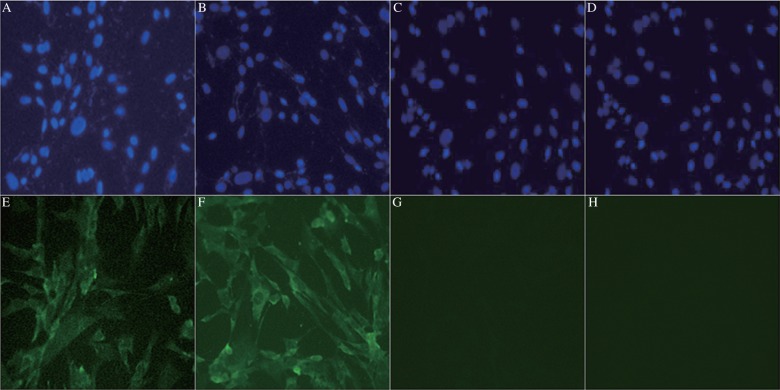
Detection of rabies virus-infected BHK-21 cells by fluorescence microscopy (400×). A, B, C and D: the cell nuclei were stained with HOECHST 33342 solution. E: rabies virus-infected BHK-21 cells stained using the 2C5 MAb. F: rabies virus-infected BHK-21 cells stained using the 3C7 MAb. G: uninfected BHK-21 cells stained using the 2C5 MAb. H: uninfected BHK-21 cells stained using the 3C7 MAb.

IFA was performed to further analyze whether the MAbs recognized viral protein in rabies-infected BHK-21 cells. The two MAbs did not show non-specific binding to uninfected cells. However, both MAbs 2C5 and 3C7 showed strong reactivity with rabies-infected cells, whereas normal mouse serum did not bind to infected cells (***Fig. 5***).

### Immunocytochemistry

**Fig. 6:**
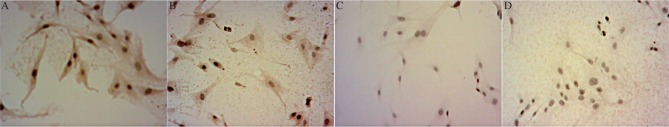
Detection of rabies virus-infected BHK-21 cells by immunocytochemistry assay. A: rabies virus-infected BHK-21 cells stained using the 2C5 MAb. B: rabies virus-infected BHK-21 cells stained using the 3C7 MAb. C: uninfected BHK-21 cells stained using the 2C5 MAb. D: uninfected BHK-21 cells stained using the 3C7 MAb.

Immunocytochemistry also indicated that the two MAbs could recognize rabies virus protein in its native form in cell samples. The results showed that infected, but not uninfected, cells were strongly stained with both anti-rabies MAbs (***Fig. 6***), while infected cells were not stained with SP2/0 supernatant (data not shown).

### AC-ELISA

**Fig. 7:**
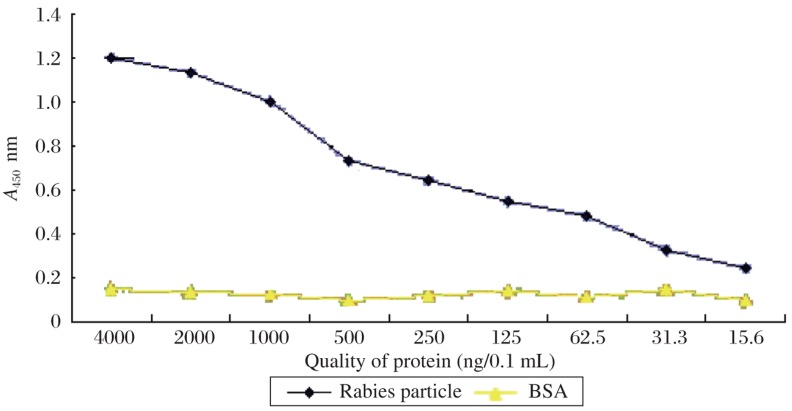
Sensitivity of AC-ELISA using MAbs. The rabies particle of strain CTN was used for quantitative analysis. BSA was used as the negetive control with the detection limit of 31.3 ng/0.1 mL.

**Fig. 8:**
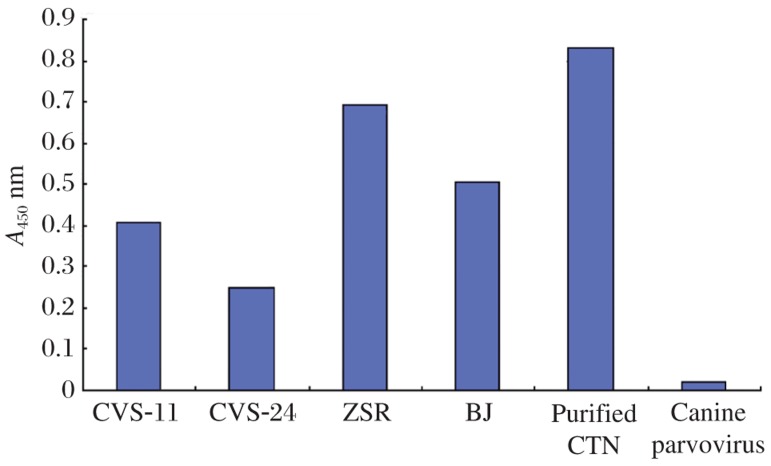
Diagnosis of mouse brain tissue samples infected with rabies virus by AC-ELISA. The samples tested were four mouse brain tissue suspension samples, one infected with rabies strain CVS-11, one with strain CVS-24 and two infected with samples from wild animals (named ZSR and BJ, no identification). Mouse brains were infected with purified virus particle of strain CTN (50 ng/0.1 mL), and canine parvovirus were used as a positive and negative control, respectively.

In order to establish a sensitive AC-ELISA for rabies virus detection, MAb 3C7, which showed the highest affinity for rabies antigen as a capture antibody, was evaluated with horseradish peroxidase-conjugated 3C7 as the detector antibody. To determine the detection limit of AC-ELISA, a serial dilution of purified rabies particles of the CTN strain was used to construct the binding curve. According to the cut-off threshold (2, which is the ratio of positive/negative), it was deduced that as little as 31.3 ng/0.1 mL of rabies protein could be detected (***Fig. 7***). To further characterize the AC-ELISA, culture supernatant (10^3.5^ TCID_50_/mL) and four mouse brain tissue suspension samples were also diagnosed as positive (***Fig. 8***). The results were confirmed by a direct fluorescent antibody test (data not shown). These results showed that 3C7 MAb could be used to detect rabies.

## DISCUSSION

Diagnosis of rabies virus antigen is conventionally done by direct FAT using rabies antinucleocapsid antibody conjugated on brain impression smears[15]. Because it is fast, reliable and inexpensive, FAT has become the choice for the diagnosis of rabies[26],[27], and is used as the standard diagnostic test for rabies virus[27]. Reverse trans-PCR and real time-PCR are too expensive or inconvenient to be applied in many developing countries. ELISA is economical, consumes fewer reagents and fits well with current facilities in many underdeveloped and developing countries where diagnostic methods are less sophisticated. A very small quantity of suspect brain material may be obtained by using a drinking straw or plastic pipette through the occipital or retroorbital route as recommended earlier[28]. This reduces any danger to the laboratory worker who conducts brain removal; in addition, materials can be transported in Eppendorf tubes containing 0.5 mL of formalin on ice.

In this study, two monoclonal antibodies specific to rabies protein were produced and charac terized by isotyping, reactivity with rabies antigens, affinity assay, dot-ELISA, immunofluorescence assay and immunocytochemistry. MAb 3C7, which showed greater affinity with antigen, was used to establish an AC-ELISA detection system.

In order to show that the antibodies were better suited for the diagnosis of rabies strains prevalent in China, the CTN strain of the rabies virus was chosen as the immunogen. The structure of the GP gene of this strain is basically stable, and the homology of the CTN strain to the street strain is higher than is either the aG or PV strain[16]. Mouse ascites or purified MAbs were used in different assays in this study. The results with both MAbs 2C5 and 3C7 were similar when either ascites or purified MAbs were used in indirect ELISA.

Among the many techniques developed for the rapid diagnosis of viral infections, AC-ELISA is a sensitive and specific method that is capable of large-scale screening in surveillance programs[30]. It also offers some advantages over the more traditional antigen detection methods[29]. Nowadays, rabies rapid enzyme immunodiagnosis (RREID) is being widely used to diagnose rabies virus[15]. However, the microplates are coated with purified rabbit anti-rabies virus polyclonal antibody, which may be less specific than a MAb-based assay. To obtain the strongest signal and the highest specificity for rabies virus detection by AC-ELISA, MAb 3C7 was evaluated because it showed stronger reactivity with virus antigen, and higher affinity with native antigen than MA6 2C5. Therefore, the use of MAb 3C7 was more likely to contribute to the high sensitivity of AC-ELISA. Four mouse brain tissue samples were examined by this AC-ELISA, and the results showed that this technique could be used in the diagnosis of clinical samples. Due to time and sample size limitations, the AC-ELISA approach has not been widely used with clinical samples. Further studies are still needed to improve the sensitivity and application to clinical specimens.

Previous research confirmed that the GP is the important antigen of the rabies virus, and it is capable of inducing and binding neutralizing antibodies (VNA) to the virus, which confer immunity against a lethal virus challenge[30],[31]. In the present study, both MAbs 2C5 and 3C7 could react with the GP (67KDa) in a Western blotting assay.

In conclusion, two MAbs specific to rabies virus protein were produced and characterized. These two MAbs could be used in dot-ELISA, IFA, immunocytochemistry and studies in rabies virus pathology. The detection limit of the established AC-ELISA re-emphasizes the sensitivity of specific MAbs for viral antigen detection, which suggests that the two MAbs will be useful for the further development of highly sensitive, easily handled and relatively rapid detection kits/tools in rabies virus surveillance, especially for the strains prevalent in China.
